# Combination of Ewing test, heart rate variability, and heart rate turbulence analysis for early diagnosis of diabetic cardiac autonomic neuropathy

**DOI:** 10.1097/MD.0000000000008296

**Published:** 2017-11-10

**Authors:** Kun Lin, Liling Wei, Zhihua Huang, Qiong Zeng

**Affiliations:** a Department of Endocrinology; b Department of Neurology, The First Affiliated Hospital of Shantou University Medical College; c Shantou University Medical College, Shantou, China.

**Keywords:** cardiac autonomic neuropathy, diabetes mellitus, Ewing test, heart rate turbulence, heart rate variability

## Abstract

The aim of this study was to compare and analyze Ewing test, heart rate variability (HRV), and heart rate turbulence (HRT) in the diagnosis of cardiac autonomic neuropathy (CAN) in diabetic patients.

Ninety diabetic patients (age 18–78) and 20 nondiabetic control subjects were studied. Standard Ewing test and 24-hour Holter was performed in all participants to evaluate CAN. Patients with Ewing score ≥2 were classified as CAN+.

The rate of CAN+ in diabetic patients [44.4% (40/90)] was higher than that in the controls [5% (1/20)] (*P* < .05). Using the HRV analysis and HRT analysis, rates of CAN+ in diabetic patients were 56.67% (51/90) and 52.22% (47/90), respectively. SD of all normal-to-normal (NN) intervals (SDNN), SD of the average NN intervals calculated over 5-minute periods of the entire recording (SDANN), low frequency power (LF), and turbulence slope (TS) were significantly correlated negatively with Ewing scores. TS (*r* = −0.68, *P* < .05) and SDNN (*r* = −0.58, *P* < .05) had the strongest correlation with Ewing scores among relevant factors. Combining TS with SDNN as diagnostic criteria for CAN, the diagnostic sensitivity can be increased to 98%.

Parameters used for evaluating parasympathetic functions in Ewing test, HR variability, and HR turbulence were found to significantly decrease in CAN+ group. The combination of SDNN and TS showed greater diagnostic value than Ewing test, HRV analysis, or HRT analysis alone.

## 1 Introduction

Cardiac autonomic neuropathy (CAN) is an important chronic complication of diabetes mellitus (DM), which can significantly increase the mortality of patients with diabetes. Early diagnosis of CAN can reduce the risks of the painless myocardial ischemia, myocardial infarction, and sudden cardiac death associated with CAN.^[1–5]^ Therefore, the American Diabetes Association (ADA) recommends that CAN diagnostic screening should be routinely performed in patients with diabetes.^[2]^ However, the diagnostic screening for CAN is not widely available due to its occult onset and its different diagnostic criteria. Ewing test is recommended as the gold standard for clinical testing, but the operation is too cumbersome. Heart rate variability (HRV) analysis is another CAN detection method recommended by the ADA, but it can be influenced by many factors.^[6–8]^ Heart rate turbulence (HRT) is a relatively new noninvasive cardiac electrophysiological method and a reliable indicator of baroreceptor sensitivity, which can reflect the regulatory function of the autonomic nervous system in the heart.^[9–11]^ At present, there is still a lack of research on the diagnostic value of these 3 methods of evaluating CAN in the diabetic patients. The objective of this study was to compare and analyze the values of these diagnostic methods for the clinical assessment of CAN in patients with DM. We hypothesized that a combination of certain parameters of both HRV analysis and HRT analysis would show higher sensitivity and specificity for the diagnosis of CAN in DM patients.

## 2 Materials and methods

### 2.1 Subjects

Patients with DM age 18 to 78 years were studied. Any subject who was pregnant, or who has been taking beta receptor blockers for the past 2 weeks, or showed signs of illnesses (e.g., arrhythmia, coronary heart disease, hepatic or renal functions tests abnormalities, tumors, psychiatric disorders, etc.) was excluded. One hundred eight patients with DM (all conforms to 1999 WHO Diabetes Diagnostic Criteria) hospitalized in the endocrinology department of The First Affiliated Hospital of Shantou University Medical College (SUMC) from November 2015 to October 2016 were recruited. Twenty-eight nondiabetic control subjects were recruited from The Health Center of SUMC during the same period. Among them, 18 diabetes patients and 8 control subjects were excluded because there were less than 2 ventricular premature beats on their 24-hour Holter recordings that would make it difficult to evaluate HR turbulence. Consequently, 90 patients and 20 nondiabetic control subjects were enrolled in the study. All participants had signed the informed consent and all procedures conformed to the Declaration of Helsinki. Patients’ blood pressure, body weight, and heart rate were recorded. Laboratory measurements including glycosylated hemoglobin (HbA1c), serum lipids including high-density lipoprotein cholesterol (HDL-C), low-density lipoprotein cholesterol (LDL-C), total cholesterol (TC), and triglyceride (TG) were investigated as well. CAN was assessed by 3 methods in each diabetic patient, including Ewing test, HRV analysis, and HRT analysis.

### 2.2 Ewing test

CAN was assessed by 5 standard cardiovascular reflex tests proposed by Ewing et al.^[12]^ Three of these tests assess parasympathetic function: heart rate responses to deep breathing (E/I ratio), to standing (30 s/15 s ratio), and to the Valsalva maneuver (Valsalva ratio). The other 2 tests assess sympathetic function: blood pressure responses to lying to standing and to a sustained handgrip. Each of these 5 tests was assigned a score of 0 for normal, 0.5 for borderline, and 1 for abnormal results, and the sum of these 5 scores made up the Ewing score, which was used to assess severity of CAN. Patients having Ewing score ≥ 2 formed the CAN+ group and patients who had Ewing score less than 2 formed the CAN− group.

### 2.3 Heart rate variability (HRV) analysis

Twenty-four hour Holter was performed in all participants to evaluate the HRV. Time-domain HRV and frequency-domain HRV indexes were analyzed. Time-domain HRV indexes include the SD of all normal-to-normal (NN) intervals (SDNN), the SD of the average NN intervals calculated over 5-minute periods of the entire recording (SDANN), percentage of adjacent RR intervals with a difference of duration greater 50 ms (PNN50, %), and the root mean square successive difference of the RR interval (RMSSD, ms). Frequency-domain HRV indexes include low frequency power (LF, ms^2^) and high frequency power (HF, ms^2^). CAN+ requires at least 2 of the following 6 abnormal parameters: SDNN <50 ms, SDANN <40 ms, RMSSD <15 ms, PNN50 <0.75%, LF <300 ms^2^, HF <300 ms^2^.^[13,14]^

### 2.4 Heart rate turbulence (HRT) analysis

HRT data were also collected from 24-hour Holter recordings. There are 2 components of HRT: turbulence onset (TO) and turbulence slope (TS). TO denotes the early acceleration phase of sinus rhythm, while TS quantitatively analyzes the late deceleration phase of sinus rhythm after premature ventricular complexes (PVCs). When T0 is greater than 0 or TS is less than 2.5 ms/RR, it is considered as CAN+.^[15]^

### 2.5 Statistical analysis

All the statistical analyses were performed using SPSS 19.0 (SPSS Inc., Chicago, IL). Data were expressed as mean ± standard deviation (SD) for continuous variables, and as percentages for categorical variables. Differences in categorical variable distribution between groups were assessed by χ^2^ test. Differences for continuous variables among the study groups were assessed by analysis of variance (ANOVA) test. Spearman rank correlation method was used to determine correlation between Ewing score and Holter variables.

## 3 Results

### 3.1 Clinical characteristics and Ewing test

Totally 90 diabetic patients (83 type 2 diabetes and 7 type 1 diabetes) and 20 nondiabetic control subjects were studied. Demographic and clinical characteristics of the study population are summarized in Table 1. Median age of diabetic patients was 54.4 years, with a median DM duration of 6.9 years. According to the criteria of Ewing test, 40 out of 90 diabetic patients (44.4%) were classified as CAN+, while the proportion in the control group was 5% (1/20). There were significant differences of Ewing scores between CAN+ and CAN− groups (2.91 ± 1.09 vs 0.57 ± 0.85, *P* < .001). Both DM duration and age were significantly higher in CAN+ group than in CAN− group (both *P* < .01).

**Table 1:**
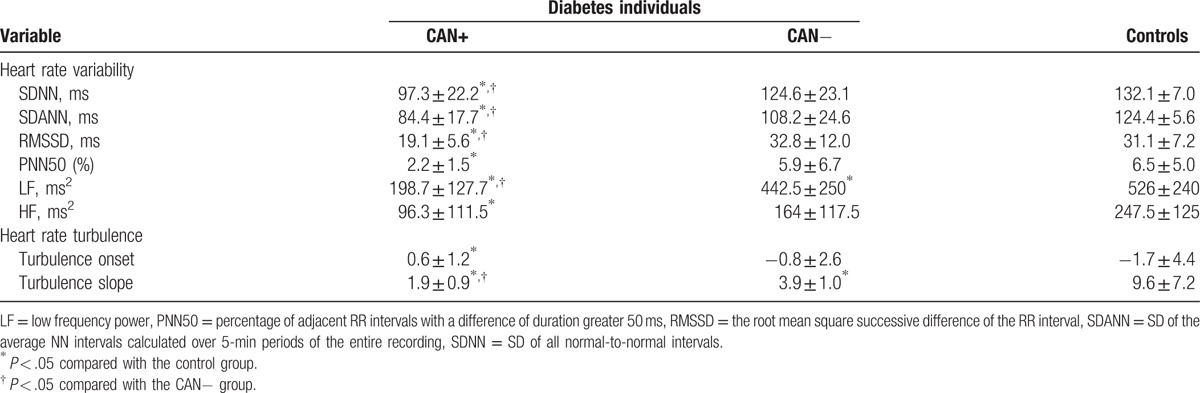
Values of Ewing test and characteristics of the study population.

### 3.2 HRV and HRT analysis

Holter data are presented in Table 2. For HRV parameters, SDNN, SDANN, LF showed significant differences among CAN+, CAN−, and control groups. SDNN, SDANN, LF in patients with CAN+ were significantly lower than patients with CAN− and control subjects. RMSSD, PNN50, and HF in patients with CAN+ were significantly lower than control subjects, but not CAN− subjects. For HRT parameters, TS significantly decreased in CAN+ group compared with individuals in CAN− and control groups. There were no significant differences of TO between CAN+ and CAN− groups but CAN+ and control groups. Using the HRV analysis and HRT analysis, rates of CAN+ in diabetic patients were 56.67% (51/90) and 52.22% (47/90), respectively.

**Table 2:**
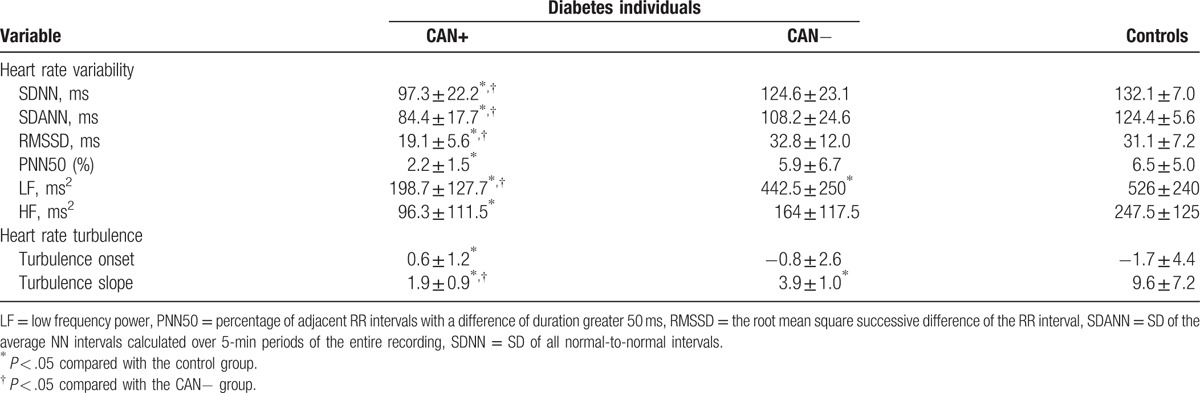
Comparison of component values of heart rate variability and heart rate turbulence parameters in diabetes patients and controls.

### 3.3 HRV, HRT parameters, and Ewing test

Correlation analyses among individuals with diabetes showed a significantly negative correlation between total Ewing scores and SDNN, SDANN, LF, and TS (Table 3). TS (*r* = −0.68, *P* < .05) and SDNN (*r* = −0.58, *P* < .05) had the strongest correlation with Ewing scores among relevant factors.

**Table 3:**
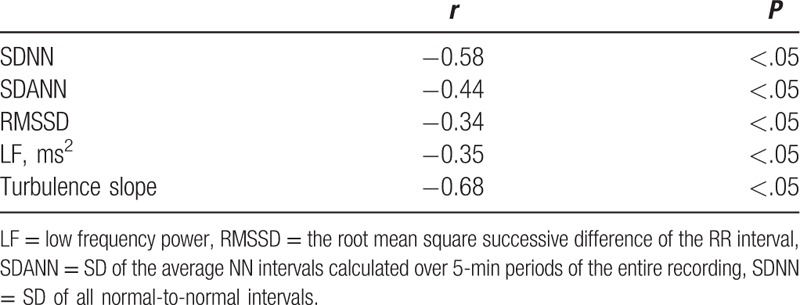
Correlation analyses between total Ewing scores and SNNN, SDANN, RMSSD, LF, and TS among individuals with diabetes.

We further explored the diagnostic value of HRV analysis and HRT analysis for the diagnosis of CAN by using the Ewing test as the gold standard (Table 4). The results showed that the sensitivity of HRV analysis and HRT analysis was similar (72% vs 75%), but the specificity of HRV analysis was much lower than HRT analysis (55% vs 65%). If TS was combined with SDNN as diagnostic criteria for CAN, the diagnostic sensitivity can be increased to 98%, the Youden index increased to 0.61, and the Kappa value increased to 0.61. It indicated that the combination of SDNN and TS showed greater diagnostic value than the HRV analysis or HRT analysis alone.

**Table 4:**
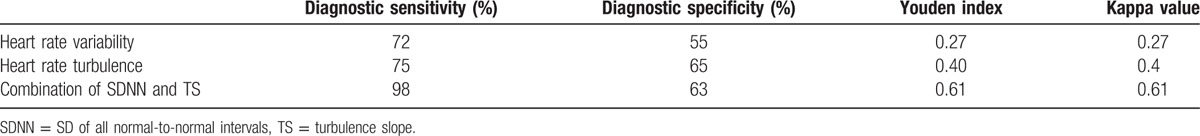
Comparison of diagnostic value among 3 diagnostic methods: HRV analysis, HRT analysis, and the combination of SDNN and TS.

## 4 Discussion

This cross-sectional study involved totally 90 diabetic patients and 20 nondiabetic control subjects. Ewing test, HRV analysis, and HRT analysis were adopted simultaneously in each of these individuals to investigate the pathologic conditions of cardiac autonomic nervous system, and to evaluate the diagnostic values of these methods for CAN. Our data revealed that the rate of CAN+ in diabetic patients was higher than that of control group by using Ewing test, HRV analysis, and HRT analysis (all >44%), of which parameters that reflect functions of parasympathetic nerves decreased significantly. HRT analysis had a similar sensitivity as HRV analysis, but the former had a higher specificity in the assessment of CAN. The most important finding was that TS and SDNN were tightly correlated with Ewing score. Significant increases in the sensitivity and the Yoden index were observed when combining SDNN and TS. Therefore, a combination of SDNN and TS showed a greater diagnostic value than using HRV analysis or HRT analysis alone, which can be a better diagnostic screening method for CAN in diabetic patients clinically.

CAN is an independent risk factor for cardiovascular and cerebrovascular diseases that accelerate the mortality rate in diabetic patients. ADA recommended that for diabetic patients, CAN should be routinely screened while taking measures to limit the associated factors. However, complicated diagnostic tests with various criteria and a lack of relevant studies make CAN become one of the diabetic complications that is most likely to be neglected. Until now, there still lacked studies concerning the diagnostic values of different analytic methods for evaluating CAN in diabetic patients. Interestingly, Weimer ^[16]^ found out that the detection rate of CAN+ when combining Ewing test and HRV indexes including RMSSD, HF, and LF was higher than that of using the HRV analysis or HRT analysis alone. It is the first time that such a clinical research was carried out on Chinese population for evaluating and analyzing CAN in DM by using Ewing test, HRV analysis, and HRT analysis. The result showed that the rate of CAN+ was significantly higher in diabetic group than the control group by using Ewing test (44.4%), HRV analysis (44.4%), or HRT analysis (52.22%). Such a relatively high rate of CAN+ in diabetic patients is of relevant clinical interest. In this study, there was also a positive correlation between CAN+ and DM duration, which was consistent with the other research findings.^[17,18]^

Ewing test and HRV analysis are comprehensive diagnostic methods for CAN, which are recommended by ADA. For Ewing test, Valsalva index, E/I and 30 s/50 s mainly represent the parasympathetic functions, while the difference between blood pressure responses to standing and supine position can assess the sympathetic functions.^[19,20]^ HRV analysis is composed of time-domain and frequency-domain parts. Time-domain HRV parameters SDNN and SDANN assess parasympathetic functions, while RMSSD and PNN50 are for sympathetic functions. Frequency-domain HRV parameter HF assesses the sympathetic functions, while LF represents both sympathetic and parasympathetic activities.^[2,11]^ Parameters of Ewing test and HRV analysis in diabetic group were significantly greater than that of control group. Of interest was the finding that parameters, including Valsalva index, E/I, SDNN, and LF of Ewing test, HRV and HRV analysis that were used for evaluating parasympathetic functions presented significant differences between CAN+ and CAN− groups. It indicated that parasympathetic dysfunctions play a major role in the development of CAN in diabetic patients. In addition, Ewing test and HRV analysis showed consistency in the autonomic function assessment.

HRT analysis has become increasingly concerned in terms of the CAN diagnosis. Balcıoğlu and Müderrisoğlu ^[1]^ found that HRT analysis was an independent predictor of CAN in type 2 diabetic patients. Our study turned out that TS significantly decreased in diabetic patients, while TO did not change markedly. The reason accounting for this phenomenon is that TO mainly reflects sympathetic activation, which prevails the parasympathetic inhibition after PVCs. On the contrary, TS is a parameter driven by parasympathetic effect, hence, influenced by CAN.

Respective merits and faults of Ewing test, HRV analysis, and HRT analysis were discussed as follows. The most classical Ewing test is of high repeatability with inexpensive assisting devices. Limitations of HRV analysis are listed: it detects only few cardiac cycles; do not necessarily reflect the actual condition of patients; has a complicated procedure; is influenced by the patient's cooperative capacity and operator's subjective factors; is analyzed semi-quantitatively; is affected by respiratory diseases and hypotensive medications, etc.^[16]^ HRV analysis, a much convenient, objective, and accurate method, can quantitatively analyze autonomic functions, and demonstrate HRV time regularity and balanced state of sympathetic and vagal effects.^[21]^ Nevertheless, lots of influencing factors diminish its specificity and stability for assessing cardiac autonomic functions and subsequently reduce its diagnostic and practical values. It is now held that HRT analysis is much more beneficial to predict the prognosis of cardiovascular diseases than the traditional methods.^[11]^ HRT is a responding result to a weak intrinsic stimulus after a PVC. On the basis of domestic and other researches, HRT can not only reflect the sensitivity of baroreflex but also the tension of autonomic nerves. It is of high sensitivity and specificity, correspondingly target at certain organs and systems. In spite of these advantages, HRT analysis can only be utilized when sinus rhythm is present and PVCs index reaches a certain value. The sensitivity and specificity of HRT analysis were higher than those of HRV analysis, as shown in our results. In addition, TS and SDNN had the tightest correlation with Ewing scores.

Some researchers considered that the relationship between SDNN, SDANN and T0, TS is correlative as well as independent. Both HRT analysis and HRV analysis can provide the quantitative information of cardiac autonomic nerves, but the physiologic contents they cover are different. Compared with HRV analysis, which mainly measures a series of physiologic reflexes to external stimulus, HRT analysis has a particular emphasis on the reflexive modulation caused by autonomic nerves following the baroreceptor activation.^[22]^ We tried to combine certain parameters of HRV analysis and HRT analysis for assessment. Most remarkably, the combination of TS and SDNN significantly increased the sensitivity and the Youden index to 0.61, revealing that the screening for CAN is highly effective by using combination of TS and SDNN.

There were few relevant researches that focus on CAN evaluation in diabetic patients through using these 3 diagnostic tests simultaneously. This research comprehensively analyzed and compared the clinical values of Ewing test, HRV analysis, and HRT analysis for assessing CAN. We figured out that TS and SDNN showed the strongest correlation with CAN+. Combining TS and SDNN together as diagnostic criteria can greatly cover the shortage, that is, low sensitivity of Ewing test. To a certain degree, this combination is beneficial to early screening for CAN in diabetic patients, providing a new clinical strategy. Nevertheless, due to a relatively small number of samples were involved in this clinical study, our hypothesis needs to be further confirmed by enlarging the sample size.

## Acknowledgment

The authors thank Dr Guoshu Yin from the Department of Medicine and UIC Cancer Center, University of Illinois at Chicago, for helpful suggestions and criticisms, and all the participants for agreeing to be in the study.
